# Increased FUN14 domain containing 1 (FUNDC1) ubiquitination level inhibits mitophagy and alleviates the injury in hypoxia-induced trophoblast cells

**DOI:** 10.1080/21655979.2021.1997132

**Published:** 2022-01-30

**Authors:** GuoQing Chen, Lu Chen, Yan Huang, XiongShan Zhu, YuanLan Yu

**Affiliations:** a Department of Obstetrics, Shenzhen Maternity & Child Healthcare Hospital, the First School of Clinical Medicine, Southern Medical University, Shenzhen, Guangdong, China; b Department of Emergency, Shenzhen Children’s Hospital, Shenzhen, Guangdong, China

**Keywords:** Preeclampsia, ubiquitination, fundc1, mitophagy, trophoblast cells, cell injury

## Abstract

Preeclampsia (PE) is a pregnancy disorder characterized by excessive trophoblast cell death. This study aims to explore the exact mechanism of the ubiquitination level of FUN14 domain containing 1 (FUNDC1) in mitophagy and injury in hypoxic trophoblast cells. In this study, HTR-8/SVneo trophoblast cells were cultured under normoxic and hypoxic conditions and PE mouse model was established. We found low ubiquitination level of FUNDC1 in hypoxic trophoblast cells and placenta of pregnant women with PE. Proteasome inhibitor MG-132 and protease activator MF-094 were added into HTR-8/SVneo trophoblast cells. Proteasome inhibitor MG-132 decreased FUNDC1 ubiquitination level while protease activator MF-094 increased FUNDC1 ubiquitination level. Inhibition of FUNDC1 ubiquitination promoted mitophagy and mitochondrial membrane potential (Δψm) in normoxic trophoblast cells, increased levels of reactive oxygen species (ROS) and malondialdehyde (MDA) and decreased levels of glutathione (GSH) and superoxide dismutase (SOD). In addition, FUNDC1 ubiquitination alleviated cell injury in PE mice *in vivo*. In conclusion, increased FUNDC1 ubiquitination level inhibited mitophagy and Δψm changes in hypoxic trophoblast cells, and thus alleviated oxidative injury.

## Introduction

Preeclampsia (PE) and eclampsia constitute two hypertensive disorders of pregnancy contributing to high incidence and death rates in pregnant women and perinatal fetuses [1]. About 3–5% of pregnant women are under the impact of PE [2]. PE often progresses silently, but in some cases it presents indicative signs including edema, weight gain, high blood pressure, and severe proteinuria [2,3]. Trophoblast cell injury is recognized as an important event in the development of PE [4]. Microparticles from hypoxic trophoblast cells can induce an intense inflammatory response in PE [5]. Dysregulated mitophagy is considered as another symbol of PE development [6]. So far, the only definitive treatment for PE has been to terminate pregnancy and deliver the neonate and placenta [7]. However, it is obvious that women who have been suffering from PE experience an increasing risk for cardiovascular and kidney disease later compared to normal women [8]. Therefore, further studies about PE are required due to the lack of effective treatment and diagnostic strategies.

Mitochondrial receptor FUN14 domain containing 1 (FUNDC1) is a mammalian mitochondrial autophagy receptor expressed in the mitochondrial outer-membrane [9] that mediates hypoxia-induced mitochondrial autophagy [10] and activates autophagy by directly binding to LC3 (mammalian Atg8 homologue) [11,12]. It is reported that mitochondrial E3 ligase membrane-associated RING-CH5 (MARCH5) inhibits hypoxia-induced mitochondrial autophagy by ubiquitinating and degrading FUNDC1 while inhibition of FUNDC1 ubiquitination and degradation improves mitochondrial sensitivity toward autophagy-induced stress [13,14]. Cell migration plays a fundamental role in embryonic development, wound repair, immune response, and tumor cell invasion and migration [15,16]. Autophagy is defined as a lysosome-dependent catabolic process where eukaryotic cells degrade long-lived proteins and their own organelles and participate in cell growth, differentiation, and homeostasis under various physiological and pathological conditions [17]. The important role of autophagy pathway has been documented in an increasing number of studies, and excessive autophagy of placental chorionic trophoblast cells will lead to trophoblast cell death [18,19]. In addition, increased autophagy of HTR-8 cells can impede cell migration and proliferation [20]. Moreover, reactive oxygen species (ROS)-dependent autophagy can induce cell pyroptosis and inhibit cell migration [21]. FUNDC1 has three TM domains and plays an important role in regulating mitophagy [11]. Under normal conditions, FUNDC1 mitophagy receptor activity is maintained through inhibition of phosphorylation by Src kinase at tyrosine 18 and by casein kinase 2 at serine 13 [22]. Under hypoxic conditions, dephosphorylation of FUNDC1 strengthens its interaction with light chain 3 (LC3), and induces the formation of isolated membranes to devour damaged mitochondria [13]. In addition, FUNDC1-mediated mitophagy protects laryngeal cancer cells against oxidative stress while FUNDC1 knockout in the liver initiates liver cancer by activating inflammation [23]. FUNDC1 plays a crucial role in hypoxia-induced mitophagy [24]. However, the role of FUNDC1 in PE is rarely studied.

Ubiquitination is a post-translational modification as well as a process of tagging a protein with ubiquitin [25]. Ubiquitination serves as an essential part in a wide variety of physiological processes, such as cell survival and innate immunity [26]. Ubiquitination can regulate both tumor-suppressing and tumor-promoting pathways in cancer and affect cancer development and progression in numerous ways [27-29]. The biological behavior of trophoblast cells in the placenta is similar to that of tumor cells. Moreover, ubiquitination participates in positive feedback regulations and induces autophagy [30]. Ubiquitinylated proteins are accumulated in PE placentas [31]. However, studies about the correlation between ubiquitination of FUNDC1 and trophoblast cell mitophagy and cell injury are rarely reported. We hypothesized that increased FUNDC1 ubiquitination level suppresses mitophagy and alleviates oxidative stress injury in PE mice. The purpose of this study was to explore the effect of different FUNDC1 ubiquitination level on mitophagy, mitochondrial membrane potential (ΔΨm), and levels of ROS, malondialdehyde (MDA), glutathione (GSH), and superoxide dismutase (SOD) in trophoblast cells in PE, along with the functional mechanism of FUNDC1 ubiquitination in oxidative stress injury by comparing FUNDC1 ubiquitination levels in placental tissues of normal pregnant women and PE patients, culturing HTR-8/SVneo trophoblast cells *in vitro* under normoxic and hypoxic conditions and establishing the PE mouse model. The study herein was implemented to testify our hypothesis and hopefully provide reference value to the prompt treatment and prognosis of PE.

## Material and methods

### Ethics statement

Ethical approval for the study was obtained from the Ethics Committee of Shenzhen Children’s Hospital. Written informed consent was obtained from each participant.

### Clinical sample collection and grouping

A total of 10 singleton pregnant women with PE and 10 healthy singleton pregnant women diagnosed in the Shenzhen Children’s Hospital were recruited. The diagnostic criteria for PE were in accordance with that of the American College of Obstetricians and Gynecologists Practice Bulletin in 2002 [32]. Singleton pregnant women with PE, excluding other pregnancy complications, were assigned to the experimental group while healthy pregnant women with no complications were assigned to the control group. Placental tissues from all participants in the two groups were collected at delivery.

### Cell culture

HTR-8/SVneo cells were purchased from Shanghai Institute of Cell Biology, Chinese Academy of Sciences (Shanghai, China) and cultured in RPMI-1640 medium (Gibco, Carlsbad, CA, USA) supplemented with 10% fetal bovine serum (FBS) at 37°C with 5% CO_2_ [33]. Cells were passaged every 2–3 days, and cells at the logarithmic phase were collected for further experiments. Cells were seeded in 6-well plates (10^5^ cells/mL, 2 mL/well). Upon 70% confluence, cells were cultured in serum-free medium for 24 h and then were assigned to 5 groups: the blank group (trophoblast cells were cultured for 24 h under normal conditions), the blank + MG-132 group [trophoblast cells were cultured for 24 h under normal conditions and added with proteasome inhibitor MG-132 (HY-13259, 25 μM, MCE, Monmouth Junction, NJ, USA)], the blank + NC group [trophoblast cells were treated with equal amount of dimethyl sulfoxide (DMSO)], the hypoxia + MF-094 group [trophoblast cells were added with 200 μmol/L COCl_2_ [34] and cultured for 24 h and then added with protease activator MF-094 (HY-112438, 120 nM, MCE)], and the hypoxia + NC group (trophoblast cells were treated with an equal amount of DMSO).

### Cell counting kit-8 (CCK-8) assay

CCK-8 kit (KeyGen, Nanjing, China) was applied to detect cell viability. HTR-8/SVneo cells in each group were seeded in 96-well plates (2 × 10^3^ cells/well) and cultured in an incubator with 5% CO^2^ at 37°C. Cell viability was recorded at 12 h, 24 h, 48 h, and 72 h. The optical density (OD) was measured using a Multiskan GO microplate reader (Thermo Fisher Scientific, Waltham, MA, USA) at 450 nm.

### Western blot

HTR-8/SVneo cells were harvested. Total protein was extracted with radioimmunoprecipitation assay buffer and quantified using the bicinchoninic acid (BCA) method. A 30-μg amount of protein was loaded and separated on sodium dodecyl sulfate-polyacrylamide gel electrophoresis and transferred to polyvinylidene fluoride membranes. After blocking, the membranes were incubated overnight at 4°C with primary antibodies FUNDC1 (ab224722, 1:500, Abcam, Cambridge, MA, USA), LC3 (ab192890, 1:2,000, Abcam), p62 (ab109012, 1:10,000, Abcam), and Beclin1 (ab210498, 1:1,000, Abcam). The following day, the membranes were taken out for balancing at room temperature and then incubated with horseradish peroxidase-labeled secondary antibody (Zhongshan Jinqiao Biotechnology Co., Ltd., Beijing, China) at room temperature for 1 h under conditions devoid of light. The membranes were washed thrice with 1xTris-buffered saline-Tween-20 (TBST). Protein bands were visualized using electrogenerated chemiluminescence and detected using Odyssey Clx Infrared Fluorescence Imaging System.

### Transwell assay

HTR-8/SVneo cells resuspended in serum-free medium were seeded in the apical chambers at a density of 1 × 10^6^ cells/mL. Medium with 20% FBS was added to the basolateral chambers. Cells were then incubated for 48 h. Cells that migrated underneath the membranes were stained and counted.

### Transmission electron microscopy (TEM)

HTR-8/SVneo cells in each group were washed with phosphate buffer saline (PBS) and centrifuged. Centrifuged trophoblast cells were collected in the centrifuge tube and fixed in 2.5% glutaraldehyde for 2 h. After washing with 0.1 mol/L phosphoric acid, trophoblast cells were fixed in 1% osmic acid fixative, dehydrated with graded ethanol, and washed with acetone. Then, trophoblast cells were embedded in acetone + embedding solution, solidified in an oven and sliced with an ultramicrotome at 50–60 nm. The slices were stained with 1% uranyl acetate and lead citrate. Transmission electron microscope (Philips, Amsterdam, the Netherlands) was employed for observation and photography.

### Detection of mitochondrial membrane potential (Δψm)

HTR-8/SVneo cells were seeded in 96-well plates (5 × 10^3^ cells/well). After appropriate treatment, cells were added with 10 μg/mL 5,5’,6,6’-tetrachloro-1,1’,3,3’-tetraethyl-imidacarbocyanine iodide (JC-1) (Beyotime, Shanghai, China) and incubated at 37°C for 20 min and washed twice with PBS, followed by detection of intensities of red fluorescence (excitation 560 nm, emission 595 nm) and green fluorescence (excitation 485 nm, emission 535 nm) using a microplate reader. The Δψm value was calculated as the ratio of JC-1 red/green fluorescence intensity. Fluorescence signal in cells were observed and recorded using fluorescence microscopy.

### Immunofluorescence staining

HTR-8/SVneo cells were seeded in polylysine-coated coverslip and washed once with PBS. Cells were fixed with 4% paraformaldehyde for 20 min. The extra paraformaldehyde was removed by 3 PBS washes. Then, the cells were permeabilized with 0.5% Triton X-100 for 10 min, blocked in 5% bovine serum albumin (BSA) for 1 h, and incubated with primary antibody LC3 (ab192890, 1:2,000, Abcam) overnight at 4°C. The next day, cells were washed thrice with PBS, and then incubated with the secondary antibody IgG (ab6785, 1:10,000, Abcam) at room temperature for 1 h. Cells were washed thrice with PBS and stained with 4ʹ,6-diamidino-2-phenylindole (DAPI). Fluorescence was observed under fluorescence microscopy (Olympus, Tokyo, Japan).

### Detection of oxidative stress-related indicators

ROS content was detected. HTR-8/SVneo cells were seeded in 96-well plates (5 × 10^3^ cells/well) and washed with PBS and then incubated with 5 mM CM-H_2_DCFH-DA (Thermo) at 37°C for 20 min without light exposure. Cells were washed twice with PBS and analyzed using a microplate reader (excitation/emission: 493/522 nm). The results were expressed in terms of ROS percentage compared to the control group.

GSH, SOD, and MDA contents were detected. HTR-8/SVneo cells were seeded in 60 nm culture dish at a concentration of 1 × 10^6^ cells/medium and washed with PBS. The supernatant was discarded after centrifugation. Cells were treated in line with the instructions of GSH Assay kit (Beyotime), SOD Assay Kit (Beyotime), and MDA Assay Kit (Beyotime). GSH, SOD, and MDA contents were detected by measuring absorbance at 410 nm, 560 nm, and 532 nm using a microplate reader.

### Establishment of PE mouse model

A total of 120 healthy C57BL/6 mice were purchased from Vital River Laboratory Animal Technology [SYXK (Shanghai), 2017–0014, Shanghai, China]. Female mice were caged with males at 1:1, and the female vaginal suppository was checked the following morning. The day the vaginal plug fell off was documented as pregnancy day 0. A total of 48 mice confirmed to be pregnant were assigned to 4 groups randomly (N = 12 in each group): the sham group, the PE group, the PE + MF-094 group, and the PE + NC group. All mice except the controls were intravenously administered with hypoxia-inducible factor 1-a (HIF1-a) adenovirus (100 μL, 8 × 10^10^ PFU/mL) via caudal vein at a slow speed on the 8^th^ day of pregnancy to establish the PE mouse model [35]. Mice in the control group were administered with the same amount of normal saline. On the 14^th^ day of pregnancy, mice in the PE + MF-094 group and the PE + NC group received subcutaneous administration of protease activator MF-094 and its negative control. Systolic pressure was measured on the 15^th^ day of pregnancy. Mouse urine was collected for detection of urine protein. The weights of the mouse fetus and placenta were measured. The placental tissues of mice were used for detection of FUNDC1 ubiquitination by co-immunoprecipitation (COIP).

### Measurement of systolic pressure and urine protein

Systolic pressure was measured on the 15^th^ day of pregnancy. Non-invasion blood pressure instrument was preheated for 15 min. Mouse tails were heated in warm water for 5 min, followed by immediate measurement of systolic pressure. The systolic pressure was measured 10 times to calculate the mean blood pressure. The whole operation should be quiet and gentle to avoid violent fluctuations in systolic pressure.

On the 15^th^ day of pregnancy, visible spectrophotometer was preheated for 30 min. Tri-distilled water, protein standard solution, and 50 μL urines in the sham group, the PE group, the PE + MF-094 group, and the PE + NC group were added separately with 3 mL coomassie brilliant blue (CBB) dye and sufficiently mixed, followed by 5-min standing. The absorbance at the wavelength of 595 nm was measured. The proteinuria concentration = standard concentration (563 mg/L) × (OD value of samples-OD value of blank well)/(standard OD value – blank OD value of blank well).

### Co-immunoprecipitation (COIP)

Clinical placental tissues were collected and cut into small scraps and made into homogenate. HTR-8/SVneo cells or placental homogenates were dissolved in Nonidet P40 lysis buffer containing 10 mM nicotinamide and 10 μM trichostatin A (TSA). The total protein was detected using Genesys 10 UV–Vis spectrophotometer (Thermo Fisher Scientific) and Pierce BCA protein assay kit (Thermo Fisher Scientific). The cell lysate was incubated with antibody FUNDC1 (1:100, Anyan, Shanghai, China) or immunoglobulin G (IgG) (ab172730, 1:100, Abcam) at 4°C overnight, respectively, and added with PureProteome™ Protein A/G Mix Magnetic Beads (Millipore Corporation, Billerica, MA, USA) and incubated for 2 h. The agarose beads were washed with cold PBS, and the binding protein was eluted. Precipitated protein was detected by Western blot using anti-Ub antibody (1:100, ab7780, Abcam) [36].

### Statistical analysis

Data were analyzed using SPSS 21.0 software (IBM Corp. Armonk, NY, USA). Normal distribution was confirmed by Kolmogorov-Smirnov. The data were presented as mean ± standard deviation. Independent sample *t* test was used to analyze pairwise comparisons while one-way analysis of variance (ANOVA) was used to analyze multi-group comparisons, followed by Tukey’s multiple comparison test. *P* value was obtained using a two-sided test. The value of *P* < 0.05 was considered statistically significant.

## Results

In this study, we first compared FUNDC1 ubiquitination level in placental tissues of normal pregnant women and PE patients, and found low ubiquitination level of FUNDC1 in placental tissues of PE patients. We then cultured HTR-8/SVneo trophoblast cells under normoxia and hypoxia, and found lower ubiquitination level of FUNDC1 in hypoxic trophoblast cells than normoxic trophoblast cells, and increased FUNDC ubiquitination level could inhibit mitophagy and ΔΨm change in hypoxic trophoblast cells, decrease ROS and MDA, and increase GSH and SOD, as well as ameliorate oxidative stress injury. Moreover, a PE mouse model was established and then the effect of increased FUNDC1 ubiquitination level on attenuation of oxidative stress injury in PE mice was validated *in vivo*.

### Ubiquitination level of FUNDC1 was low in placental samples of PE patients

**Figure 1.**
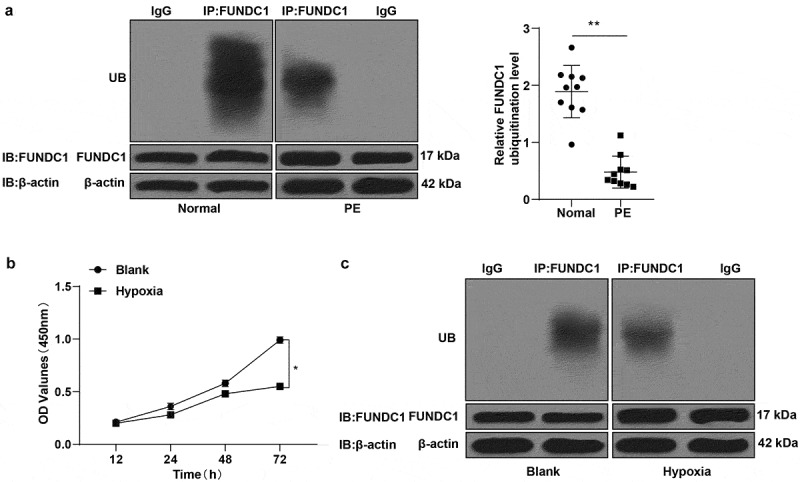
Ubiquitination level of FUNDC1 was low in placental samples of PE patients. A: the ubiquitylation levels of FUNDC1 in normal pregnant women and pregnant women with PE were detected by COIP; B: proliferative ability of trophoblast cells was detected by CCK-8; C: the ubiquitination levels of FUNDC1 in normoxic and hypoxic trophoblast cells were detected by COIP. The experiment was repeated three times. Data were expressed as mean ± standard deviation. Data in panel B were analyzed by independent sample *t* test, *P < 0.05.

Several studies have reported high expression of FUNDC1 in malignant tumors such as breast cancer and cervical cancer [37,38]. The ubiquitination and deubiquitination process is closely associated with tumorigenesis [39]. The biological behavior of trophoblast cells in the placenta resembles tumor cell behavior. Therefore, we hypothesized a correlation between ubiquitination levels of FUNDC1 with PE. Firstly, the FUNDC1 ubiquitination levels in placenta between normal women and pregnant women with PE were determined by COIP, and the result showed that the FUNDC1 ubiquitination level in pregnant women with PE was lower than that in normal pregnant women (Figure 1a). To further explore the role of FUNDC1 in trophoblast cells, HTR-8/SVneo cells were cultured under hypoxic and normoxic conditions. CCK-8 assay was applied to detect the proliferative ability of trophoblast cells, and the result revealed that the proliferative ability of trophoblast cells in the hypoxia group decreased significantly at 48 h and 72 h compared to that in the blank group (*P* < 0.05, Figure 1b).

Moreover, the ubiquitination level of FUNDC1 in trophoblast cells under normoxic and hypoxic conditions was detected by COIP. The result indicated that the ubiquitination level of FUNDC1 in trophoblast cells cultured under hypoxic condition was lower than that under normoxic condition (Figure 1c).

### Increased ubiquitination level of FUNDC1 inhibited mitophagy in hypoxic trophoblast cells

**Figure 2.**
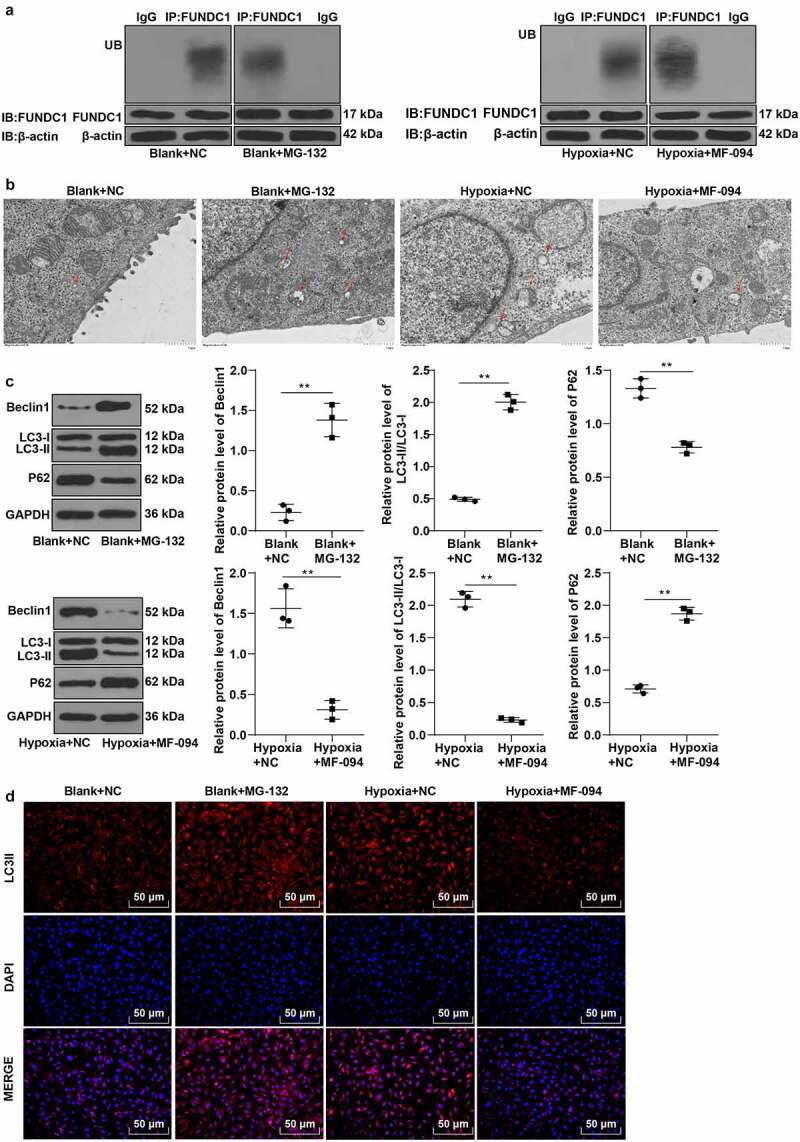
Increased ubiquitination level of FUNDC1 inhibited mitophagy in hypoxic trophoblast cells. Proteasome inhibitor MG-132 and protease activator MF-094 were added to HTR-8/Svneo trophoblast cells under normoxic and hypoxic condition to inhibit or promote ubiquitination level of FUNDC1. A: the ubiquitination levels of FUNDC1 in trophoblast cells in the NC, MG-132 and MF-094 group were detected by COIP; B: autophagosomes in each group were observed under the TEM; C: expressions of LC3-II, LC3-I, p62 and Beclin1 were determined by Western blot; D: fluorescence expression of LC3-II was observed by immunofluorescence staining. The experiment was repeated three times. Data were analyzed using independent sample *t* test, **P < 0.01.

As a novel receptor protein of mitochondria in mammalian cells, FUNDC1 is capable of inducing mitophagy under hypoxic condition [40]. Studies have reported that the disruption of FUNDC1 activation can abate mitophagy [41,42]. To further investigate the effect of FUNDC1 ubiquitination on mitophagy in hypoxic trophoblast cells, proteasome inhibitor MG-132 was added into normoxic trophoblast cells while protease activator MF-094 was added into hypoxic trophoblast cells to reduce or increase the ubiquitination level of FUNDC1 (*P* < 0.01, Figure 2a).

TEM showed an increase of autophagosomes in the blank + MG-132 group compared to the blank + NC group and a reduction of autophagosomes in the hypoxia + MF-094 group compared to the hypoxia group (*P* < 0.01, Figure 2b). The expressions of LC3-II, LC3-I, p62, and Beclin1 were detected by Western blot and the result indicated that the ratio of LC3-II/LC3-I and expression of Beclin1 were increased while p62 expression was decreased in the blank + MG-132 group (all *P* < 0.01, Figure 2c), when the conditions were opposite in the hypoxia + MF-094 group (all *P* < 0.01, Figure 2c).

Immunofluorescence staining showed that the fluorescence expression of LC3-II was increased in the blank + MG-132 group but significantly decreased in the hypoxia + MF-094 group (*P* < 0.01, Figure 2d).

The results above demonstrated that low ubiquitination level of FUNDC1 promoted autophagy while high ubiquitination level of FUNDC1 inhibited autophagy.

### Increased ubiquitination level of FUNDC1 inhibited Δψm change

**Figure 3.**
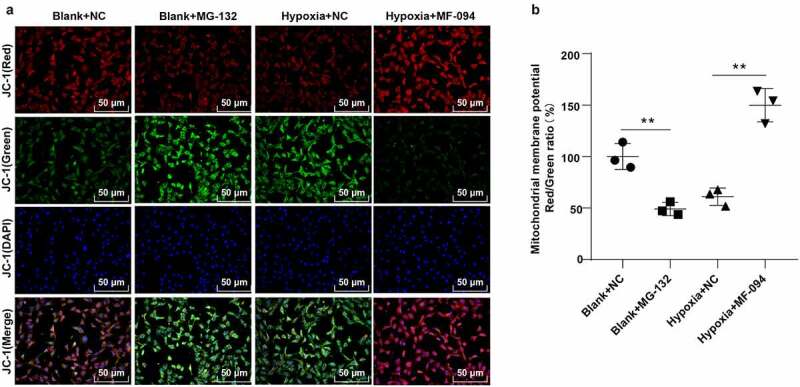
Increased ubiquitination level of FUNDC1 inhibited Δψm. A: fluorescence detected under a fluorescent microscopy in cells with JC-1. JC-1 (red) represented red fluorescence detected at 560 nm excitation and 595 nm emission. JC-1 (green) represented green fluorescence detected with at 485 nm excitation and 535 nm emission. JC-1 (Merge) was the overlapping image of JC-1 (red) and JC-1 (green); B: ratio of red and green fluorescence intensities detected by microplate reader. The experiment was repeated three times. Data were analyzed using one-way ANOVA, followed by Tukey’s Multiple comparisons test, **P < 0.01.

The loss of Δψm induced by the inhibition of mitochondrial complex I activity is considered as an important step at the beginning of apoptosis. In normal mitochondria, polymers were formed in mitochondrial matrix by accumulating JC-1 and producing strong red fluorescence while JC-1 was presented as monomers in cytoplasm and produced green fluorescence due to the decrease or loss of Δψm in unhealthy mitochondria [43]. As shown in Figure 3a-B, compared to the control group, the green fluorescence was enhanced, and Δψm was decreased in the blank + MG132 group (*P* < 0.01). The red fluorescence was strengthened and Δψm was increased in the hypoxia + MF-094 group (all *P* < 0.01). These results suggested that decreased ubiquitination level of FUNDC1 induced abnormal Δψm.

### Increased ubiquitination level of FUNDC1 attenuated injuries of hypoxic trophoblast cells

**Figure 4.**
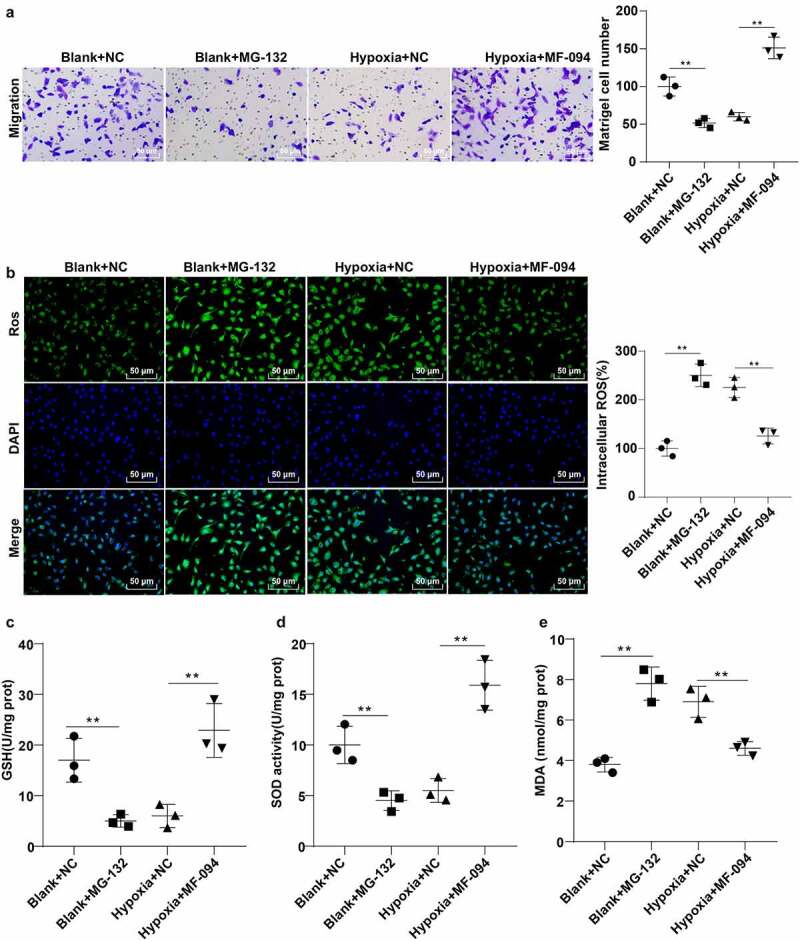
Increased ubiquitination level of FUNDC1 attenuated injuries of hypoxic trophoblast cells. A: Number of migrated cells in hypoxic condition was counted by Transwell assay; B: fluorescence probe DCFH-DA staining was performed to detect the expression of ROS; C-E: contents of GSH, SOD and MDA were detected by colorimetry. The experiment was repeated three times. Data were analyzed using one-way ANOVA, followed by Tukey’s Multiple comparisons test, **P < 0.01.

Abnormal migration of trophoblast cells represents the main pathogenesis of PE [44]. To explore the effect of FUNDC1 ubiquitination on hypoxia-induced cell injury, Transwell assay was applied to count the migrated hypoxic trophoblast cells. The result suggested that the number of migrated hypoxic trophoblast cells was reduced in the blank + MG-132 group but increased in the hypoxia + MF-094 group (*P* < 0.01, Figure 4a).

Excessive ROS is a major contributor to cell injury [45]. Thereby, fluorescence probe dichlorodihydrofluorescein diacetate (DCFH-DA) was utilized to detect ROS content. The result showed that the fluorescence intensity for ROS content was enhanced in the blank + MG-132 group and reduced in the hypoxia + MF-094 group (all *P* < 0.01, Figure 4b).

The contents of GSH, SOD, and MDA were measured by colorimetry. The contents of GSH and SOD were decreased (*P* < 0.05), while MDA content was increased in the blank + MG-132 group (*P* < 0.05, Figure 4c–4e). However, the trend was reversed in the hypoxia + MF-094 group (*P* < 0.01, Figure 4c–4e). These showed that increased FUNDC1 ubiquitination level attenuated hypoxia-induced injuries in trophoblast cells.

### Increased ubiquitination level of FUNDC1 alleviated cell injury in PE mice

**Figure 5.**
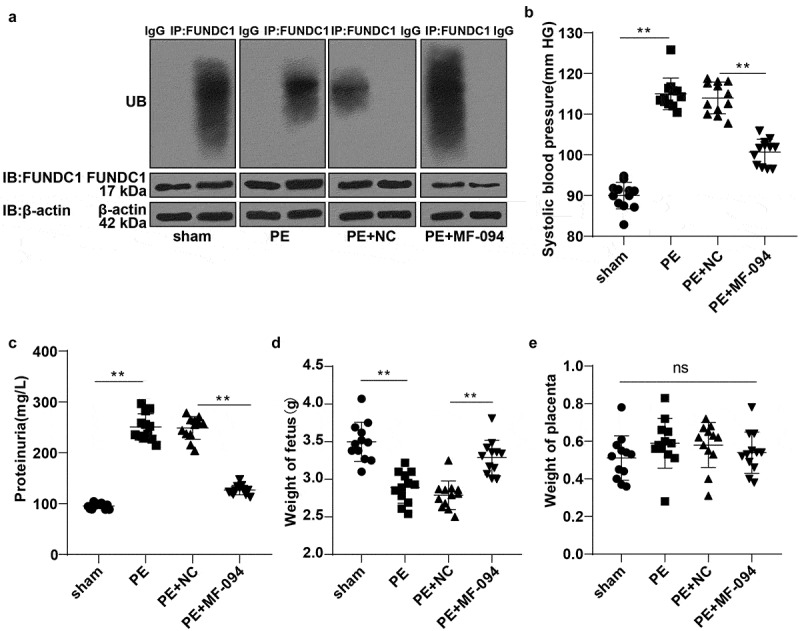
Increased ubiquitination level of FUNDC1 alleviated cell injury in PE mice. A: the ubiquitination levels of FUNDC1 in each mice were detected by COIP, N = 12; B-C: tail artery systolic pressure and urine protein were detected by tail-cuff and CBB method, N = 12; D-E: the weight of animal fetus and placenta was measured, N = 12. Data were analyzed using one-way ANOVA, followed by Tukey’s Multiple comparisons test, **P < 0.01.

To explore the *in vivo* mechanism of FUNDC1 ubiquitination level in mitophagy and cell injury, PE mouse model was established. The ubiquitination level of FUNDC1 was low in PE mice. We therefore injected protease activator MF-094 into mice subcutaneously to promote the FUNDC1 ubiquitination (*P* < 0.01, Figure 5a). Tail artery systolic pressure and urine protein were detected using the tail-cuff method and CBB method. Compared to the sham group, the systolic pressure and urine protein in PE mice were increased significantly (*P* < 0.05). On the 15^th^ day of pregnancy, the systolic pressure and urine protein were decreased in the MF-094-treated PE mice (*P* < 0.01, Figure 5b and 5c). Animal fetus and placenta were weighed. The average weight of PE fetus was reduced (*P* < 0.05), and the average weight of fetus in the PE + MF-094 group was increased (*P* < 0.01, Figure 5d). However, there was no statistically significant difference in placenta weight among normal mice, PE mice, and MF-094-treated mice (*P* > 0.05, Figure 5e).

These results indicated that increased FUNDC1 ubiquitination level alleviated trophoblast cell injury in PE mice.

## Discussion

PE remains one of the main reasons for poor perinatal outcomes owing to impaired placental development [46,47]. FUNDC1 ubiquitination is a process where mitochondria were desensitized to prevent unnecessary removal of mitochondria via mitophagy [13]. In this study, we explored the mechanism of FUNDC1 ubiquitination level on mitophagy and cell injury in hypoxic trophoblast cells and placental tissues of PE patients. The result showed that increased FUNDC1 ubiquitination level inhibited mitophagy and Δψm and further attenuated cell injury in PE.

FUNDC1 can induce mitophagy to protect mitochondria [48]. PLK1 has been reported to induce mitophagy via modulation of the AMPK/FUNDC1 pathway [49]. Overexpression of FUNDC1 is adequate for mitophagy of a number of cancer cell lines [38]. Placental trophoblast cells such as malignant tumor cells rapidly invade maternal tissues in the early stages of pregnancy [50]. According to a previous study, δ-tocotrienol can impair the viability of trophoblast cells, induce apoptosis, repress cell growth and migration, and thus ameliorate PE [51]. What is more, inhibition of PROX1-AS1 restrains PE progression by repressing apoptosis and facilitating migration and invasion of HTR-8/SVneo trophoblast cells [52]. We therefore speculated an association between FUNDC1 and trophoblast cells of PE pregnant women. In the current study, the ubiquitination level of FUNDC1 was lower in PE pregnant women than that in normal pregnant women. The lack of invasiveness in trophoblast cells can result in incomplete remodeling of spital artery and consequently placental ischemia and hypoxia, oxidative stress, and systemic inflammatory response during placenta implantation [53]. To further explore the role of FUNDC1 in trophoblast cells, HTR-8/SVneo cells were cultured under hypoxic and normoxic conditions. The proliferative ability of hypoxic trophoblast cells was decreased significantly at 48 h and 72 h and ubiquitination level of FUNDC1 in hypoxic trophoblast cells was lower than that in normoxic trophoblast cells. Accordingly, the result of our study showed that FUNDC1 ubiquitination level was low in placental tissues of PE patients and ubiquitination level of FUNDC1 might be associated with the development of PE and the proliferation of hypoxic trophoblast cells.

Previous study found FUNDC1 involvement in mitophagy via interaction with and recruitment of LC3 to mitochondria [54]. To further investigate the effect of FUNDC1 ubiquitination on mitophagy in hypoxic trophoblast cells, proteasome inhibitor MG-132 was added into normoxic trophoblast cells while protease activator MF-094 was added into hypoxic trophoblast cells. The result showed that the expressions of autophagy-specific markers LC3-II/LC3-I and Beclin1 were increased after adding proteasome inhibitor MG-132 in normoxic trophoblast cells while hypoxic trophoblast cells added with protease activator MF-094 presented the opposite tendency. According to Chen et al., MARCH5 fine-tunes hypoxia-induced mitophagy by regulating the ubiquitin-mediated degradation of FUNDC1 [13]. Silencing FUNDC1 attenuates chronic obstructive pulmonary disease by inhibiting mitochondrial autophagy [55]. It is well-known that knockdown of endogenous FUNDC1 significantly prevented hypoxia-induced mitophagy [10]. Therefore, decreased ubiquitination level of FUNDC1 promoted mitophagy while increased ubiquitination level of FUNDC1 inhibited mitophagy.

The inhibition of mitochondrial apoptosis pathway and the enhancement of mitochondrial function can alleviate PE symptoms [56,57]. Δψm dissipation might contribute to vascular endothelial dysfunction in PE [58]. In addition, FUNDC1 is an outer mitochondrial membrane protein [41] and Δψm is an indicator for apoptosis [59]. Our results showed that the green fluorescence was enhanced and Δψm was increased in trophoblast cells added with MG-132 while the red fluorescence was strengthened and Δψm was decreased in hypoxic trophoblast cells added with MF-094. The result is consistent with one recent study showing that the FUNDC1 knockdown restores Δψm loss [60]. And FUNDC1-related mitophagy sustains Δψm [61]. Taken together, decreased ubiquitination level of FUNDC1 induced Δψm change.

The imbalance of ROS can lead to vasodilatory dysfunction in PE [62]. Insufficient trophoblast invasiveness/migration is one major cause of PE [63]. It is important to eliminate excessive ROS for the survival of trophoblast cells because mitochondrial damage can result in apoptosis [35]. In our study, we found that hypoxic trophoblast cell migration was promoted and ROS production was declined after addition of MF-094. GSH and SOD content were both increased while MDA content was decreased in hypoxic trophoblast cells added with MF-094. Previous study has also shown that the accumulation of ROS can cause cell damage, reduce intracellular SOD synthesis, and decrease SOD activity in placental tissues [64]. Low levels of FUNDC1 are associated with the expression of genes involved in ROS signal transduction and metastasis (cell migration/invasion) [65]. As a result, our study revealed that increased ubiquitination level of FUNDC1 attenuated injuries in hypoxic trophoblast cells.

PE is characterized by severe hypertension and proteinuria [66]. To further explore the mechanism of FUNDC1 ubiquitination level cell injury, a PE mouse model was established. We found low ubiquitination levels of FUNDC1 in PE mice. Then, protease activator MF-094 was injected subcutaneously to promote the ubiquitination level of FUNDC1. In this study, the result showed that increased systolic pressure and urine protein in PE mice were reduced significantly after the injection of MF-094. The average weight of fetus of PE mice was reduced but was increased after injection of MF-094. Artery blood pressure is an important predictor of PE [67]. The quantity and quality of urine proteins are important factors in evaluating PE severity [68]. One major consequence of PE is low birth weight fetus [69]. These results suggested that increased ubiquitination level of FUNDC1 alleviated trophoblast cell injury in PE mice.

In conclusion, this study has revealed that enhanced FUNDC1 ubiquitination could alleviate trophoblast cell mitophagy and injury, which may provide reference value for early PE diagnosis and potential target for PE treatment. However, the direct function of FUNDC1 ubiquitination in the treatment of PE has not been discovered yet. Further studies are required to explore the effectiveness of FUNDC1 ubiquitination level on PE.

## Conclusion

FUNDC1 ubiquitination level was low in both the placental tissues of PE patients and hypoxic trophoblast cells. Increased FUNDC1 ubiquitination level could inhibit mitophagy and ΔΨm change in hypoxic trophoblast cells, decrease cellular ROS and MDA, increase GSH and SOD, and mitigate cell injury in hypoxic trophoblast cells and PE mice.

## Data Availability

All the data generated or analyzed during this study are included in this published article.
